# The Use of Formative Assessment in Postgraduate Urology Training: A Systematic Review

**DOI:** 10.7759/cureus.27162

**Published:** 2022-07-22

**Authors:** Rehan N Khan, Nadeem A Siddiqui

**Affiliations:** 1 Urology, Al-Amiri Hospital, Kuwait City, KWT; 2 Surgery, Aga Khan University Hospital, Karachi, PAK

**Keywords:** formative feedback, medical education, formative assessments, post-graduate training, urology

## Abstract

Formative assessment is an essential component of surgical training. However, it is not usually a mandatory component in postgraduate curricula. The purpose of this study is to identify and evaluate how formative assessments are integrated into postgraduate urology training in programs across the globe. This study consisted of a systemic review to see how formative assessments are being implemented in various urology programs globally. A total of 427 articles were identified for the literature review. Of these, only 10 were included and critically appraised. These studies explored various techniques for exploration of formative assessments in urology training programs, which included established tools, such as portfolio reviews, and direct observations of procedure skills (DOPS); novel tools, including the Dutch urology practical skills (D-UPS) program and Ottawa surgical competency operating room evaluation (O-SCORE); and curricular models. Nine of the 10 articles favored their potential utility in formative assessments. Current literature involving formative assessments in postgraduate urology programs is scarce, and available resources have a high heterogeneity between them. More structured formative assessments need to be incorporated into surgical training programs, and affiliated training institutions should be encouraged to integrate them into their curricula.

## Introduction and background

Background

Assessment is fundamental to the education process, yet is never without a specified purpose. Summative assessments are often high stakes and are conducted with the intent to demonstrate learning. Likewise, their utility is mainly focused on decision-making and promotions. On the other hand, formative assessments are designed to facilitate learning and offer the unique opportunity to impart highly specified and individualized feedback to the learner, with the intent to improve the quality of learning through any given activity [1,2]. Feedback can help identify gaps in knowledge and/or skills as well as clarify course objectives and expectations, thus aiding in the student’s understanding and assisting in guiding the direction of their efforts and time [3,4]. Additionally, as formative assessments are usually done in a non-threatening and non-high-stakes environment, they provide the learner an opportunity to demonstrate their knowledge and prowess in a less stressful environment, which has been shown to help in the development of professional identity and, in turn, confidence in making important clinical decisions [1,5].

Efforts must also be taken to ensure that the learning environment is conducive to more individualized feedback, irrespective of the performance of one’s peers, yet more in line with the set standards and goals of the program [6,7]. In most cases, it is not merely a matter of the presence of feedback in a program but rather the quality of feedback offered. "Quality" depends upon several factors, including the timing, the contextual background, and its structure and format [7]. Another issue is whether the learner can properly interpret the feedback being delivered. Failure to make the correlation between criticism and practical application may, in turn, lead to demotivation [8,9].

Over the years, there has been an increased interest in formative assessment in surgical training. However, these efforts have mostly been directed toward the development of particular skills in certain settings, for example, in the application/development of scoring tools such as generic operative supervised learning event (GOSLE), objective structured assessments of technical skills (OSATS), and direct observation of procedural skills (DOPS), and for simulators [10-12]. Another concept that has been recently developing is the integration of workplace assessments in the confines of medical training, which has been shown to serve a primarily formative function [13]. Though these assessments have been shown to promote learning, such activities are more often used with summative intent [13-16].

Despite the obvious benefits of formative assessment in a structured training program, it is not usually a mandatory component of curricula, especially in developing countries, like Pakistan [17], where more emphasis is often aimed toward summative evaluations. The purpose of this study is to identify and examine the various means through which formative assessments have been integrated into postgraduate urology training, through a systematic review of literature, to generate recommendations as to how to improve or implement a more structured "formative" component to the training curriculum.

Methodology

This is a systematic literature search to gauge the various avenues through which formative assessment had been applied in urology training, in literature. Databases including MEDLINE (PubMed and PubMed Central), EBSCO CINAHL Plus, Wiley Cochrane Library, and ProQuest Theses & Dissertations were used. This section of the review followed the PRISMA (Preferred Reporting Items for Systematic Reviews and Meta-Analysis) model [18]. The search parameters/keywords included "formative," "urology," and "residency."

Studies (original articles only) pertaining to postgraduate training programs and relating to trainee assessments were included. However, review articles, case reports, and papers focused on clinical diseases or specifically only relating to surgical techniques and simulation were excluded. Searches were not limited by language. The abstracts (and full texts) of these articles were screened to determine their relevance to the topic and identify emerging themes.

## Review

Results

The primary aim of this literature review is to identify the spectrum of formative assessment strategies being applied by various urology training centers across the globe, within their programs. Studies were identified that investigated the effectiveness of various assessment tools, which may be applied to formative evaluations. Due to the relative scarcity of works on this specified matter, interestingly, the search was unable to find any primary study that directly asked this question.

**Figure 1:**
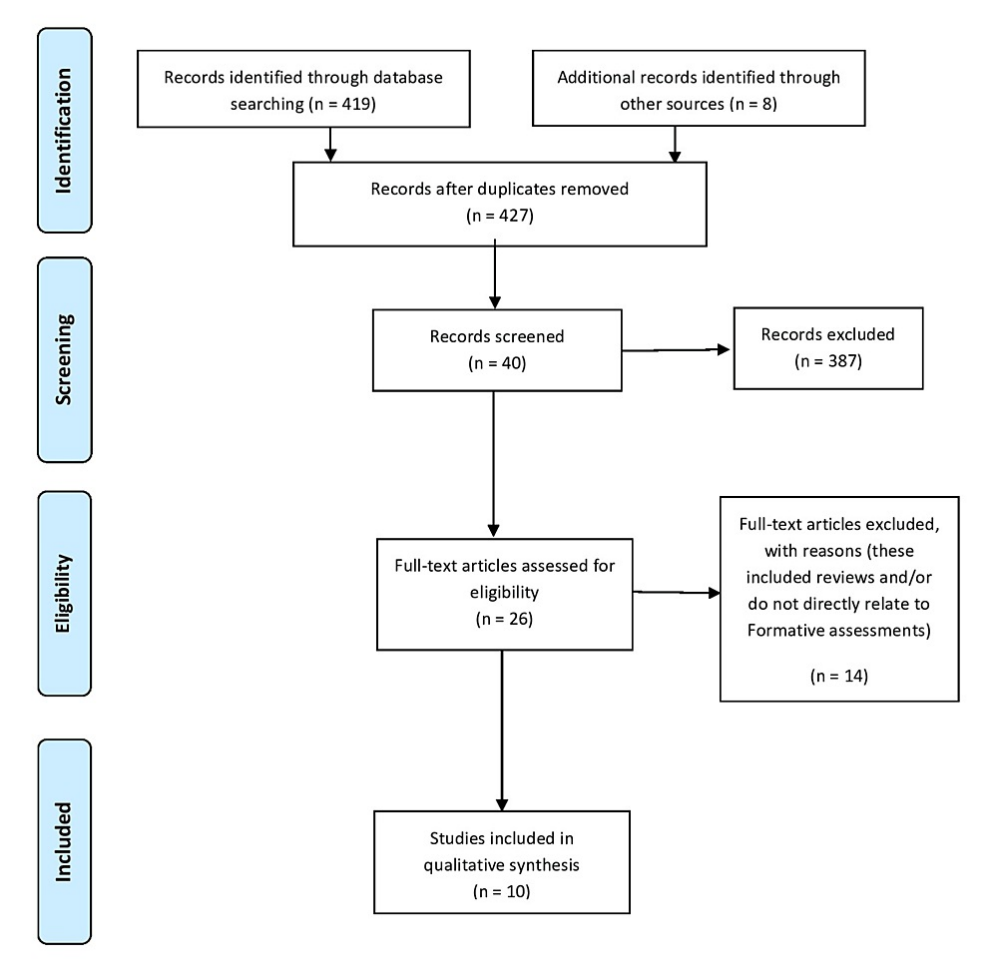
PRISMA flow diagram PRISMA: Preferred Reporting Items for Systematic Reviews and Meta-Analyses.

A total of 427 papers were initially identified through the database search. Out of these, only three duplicates were found. After screening, the full texts of 40 articles were deemed to be relevant to the main objectives. Several of these studies were rejected as they were reviews or did not include any primary research. Furthermore, studies that did not directly align with formative evaluations or directly involve postgraduate training in urology were also excluded. Ultimately, only 10 articles were found to meet the inclusion criteria and were thus included in this review. Of these 10 selected articles, half were cross-sectional studies, and the other half was based on questionnaires. Due to the discordance between the study designs and the parameters being evaluated in each individual paper, meta-analysis was not possible (Figure 1).

The studies included in this review looked at the training program from varying perspectives. The majority were conducted from the point of view of the trainees, but studies by Sebesta et al. [19], Fitzpatrick et al. [20], Aydin et al. [21], and Sibert et al. [22] considered the faculty perspective, while de Vries et al. [23] and Alkhayal et al. [24] included program directors. The number of participants varies between the studies.

On review of these articles, three common themes depending on the tools/strategies being assessed were observed. They were (1) established tools (DOPS, problem-based assessment [PBA], OSATS, etc.), (2) novel techniques (Ottawa surgical competency operating room evaluation [O-SCORE], nontechnical skills for urological surgeons [NoTSUS], and Dutch urology practical skills [D-UPS]), and (3) training models (Table 1).

**Table 1:** Results of the systemic review *Study focused more on the quality of the assessments and how it influences feedback DOPS: Direct observation of procedural skills; D-UPS: Dutch urology practical skills; EMQ: Extended match questions; NoTSUS: Nontechnical skills for urological surgeons; OSATS: Objective structured assessment of technical skill; O-SCORE: Ottawa surgical competency operating room evaluation; PBA: Problem-based assessment; SCTs: Script concordance tests; PD: Program directors.

Author’s Name	Year of Publication	Country of Origin	No. of Participants	Competencies Assessed	Strategies Assessed/Described	Study Type	Outcomes (Usefulness of Model in Formative Evaluations)
Fitzpatrick et al. [20]	2019	Canada	18 residents, 13 faculty	Technical competencies	O-SCORE	Survey	Useful
McDougall et al. [30]	2007	USA	8 residents	Technical competencies	OSATS	Cross-sectional	Useful
Ali et al. [25]	2019	Pakistan	20 residents	Technical competencies	DOPS	Cross-sectional	Useful
Sebesta et al. [19]	2019	USA	38 residents	Nontechnical competencies	Milestones system	Survey	Not useful
Ali et al. [26]	2012	UK	170 residents	Technical competencies	DOPS PBA	Cross-sectional	Useful*
Aydin et al. [21]	2020	UK	43 residents, 19 faculty	Nontechnical competencies	NoTSUS	Survey	Useful
de Vries et al. [23]	2015	The Netherlands	87 residents, 45 PDs	Both	D-UPS	Survey	Useful
Alkhayal et al. [24]	2012	Saudi Arabia	71 PDs	Both	OSATS, hand-motion analysis, portfolio, videotape analysis	Survey	Useful
Sibert et al. [22]	2006	France	42 residents, 61 faculty, 10 students	Nontechnical, clinical reasoning, problem-solving, data interpretation	SCTs	Cross-sectional	Useful
Nazim et al. [27]	2019	Pakistan	9 residents, 6 interns, 12 students	Nontechnical, clinical reasoning, problem-solving, data interpretation	SCTs EMQ	Cross-sectional	Useful

Established Tools

Five of the identified studies were focused on already established and commonly used assessment tools. Ali et al. (2019) [25] and Ali et al. (2012) [26] both focused on DOPS; Sibert et al. [22] and Nazim et al. [27] explored the script concordance test (SCT), whereas Alkhayal et al. [24] examined the utility of multiple strategies including OSATS, portfolio review, and videotape and hand-motion analyses.

A commonality between the studies that focused on DOPS is that they focused primarily on technical/surgical competencies, and had both been cross-sectional studies. Ali et al. [25] focused their assessments on three basic urological procedures, namely, cystoscopy, transurethral resection of the prostate (TURP), and ureterorenoscopy (URS)), and found a statistical improvement in the assessment scores (p = 0.04) before and after DOPS. On the other hand, Ali et al. [26] focused more on the quality of the feedback being given in DOPS as well as PBAs. These authors found that qualities like positive language/encouragement in feedback, including the trainees’ strengths in the feedback, and offering suggestions and explanations were associated with better responses in the trainees.

In their study, Alkhayal et al. [24] explored assessment strategies in multiple surgical fields, with urology being only one of them. It was observed that portfolio review and videotaped analysis of trainees were the most popular strategies among the program directors in urology. Among the program directors included in this study, the vast majority (79%) found that portfolio reviews were useful in offering formative feedback, whereas only 19% were neutral on its benefit. Sixty-three percent of the program directors in urology found videotaped analysis a useful tool and advocated its use in common practice. The authors described that nearly 79% of the programs were using this modality as an important assessment tool in practice and advocated its use.

SCTs give the unique opportunity to assess clinical reasoning, data interpretation, and correlation of this information toward appropriate treatment options. Studies by Nazim et al. [27] and Sibert et al. [22] found that this test was a good tool to distinguish the level of the learner and was found potentially useful in triggering the thinking process, thus can be an effective tool in formative evaluations. However, one limitation was the time and effort required in constructing these questions.

Novel Techniques

Three of the identified articles explored novel techniques. All these three articles were questionnaire-based studies, which included perspectives from trainees and faculty/program directors.

The D-UPS program was described by de Vries et al. [23]. This program is a one-hour structured program, which had been constructed with formative evaluations in mind, with only eight sessions constructed at the time. Each session consists of a brief online formative test, followed by a discussion on the contents of the test, and then a skills demonstration and follow-up assessment. The authors found 92% and 87% approval ratings among residents and faculty, respectively, as it encouraged a global evaluation of the trainee and was designed to assess for both technical and nontechnical competencies. This model of teaching and assessment has a potential for standardized use and may help bridge the gaps between the knowledge base of a trainee and the current guidelines. The main limitation was that more senior trainees found it harder to adapt to this new strategy.

Fitzpatrick et al. [20] explored the O-SCORE, a previously validated tool [28,29] that has been designed to provide timely feedback on the surgical competencies of the trainee. In their article, the authors explored the benefits of having a web-based O-SCORE tool, which may be potentially linked to the student’s portfolio and also linked to an alert system, thus promoting timely feedback and avoiding memory bias. This computerized format was found to be easy to use and readily available. Furthermore, it was also observed that physical feedback from the faculty has also increased, and the trainee’s progress could be tracked more objectively. Around 81% of the trainees felt a positive impact of the O-SCORE on their training.

Lastly, in their article, Aydin et al. [21] described the NoTSUS (nontechnical skills for urological surgeons). These "encounters" chiefly involved two-hour training blocks, followed by a feedback session focused on, as the name suggests, the nontechnical aspects of training. This included communication and interpersonal skills and how to minimize the influence of potential distractors in the operating room. The authors described four key scenarios in which these role-playing sessions had been designed. These include faulty debriefing at the start of the procedure, faulty instruments, an inexperienced scrub nurse, or even intra-operative shock. Each session is videotaped and then used in the eventual feedback session. This was generally well-received by residents and students, alike. Statistically, they found that improvement had been observed between the first and third sessions (p = 0.04) but not in any subsequent sessions.

Training Models

Two of the selected studies were directed toward training models. However, similarities between the two studies end there. Sebesta et al. [19] conducted a questionnaire-based survey among program directors on the use of the Milestones system in urology training programs across the United States. The Milestones system assesses six key competencies, such as patient care, communication skills, system-based practice, professionalism, medical knowledge, and problem-based learning and improvement, through 34 further subdomains in urology. Structurally, these competencies are to be assessed twice per year. However, despite the frequency of these assessments, a lot of faculty felt that scores in the Milestones domains did not correlate well with either in-service exams (58%) or boards (49%). Interestingly, this is the only study among the search results which was concluded to be not useful in the evaluation of trainees.

McDougall et al. [30] described their four-year residency curriculum that incorporated the various sections of Campbell’s urology, via a rigid year-wise 12-monthly topic schedule, and shared the results of their annual in-service examinations before and after the integration of this curricular design. According to their curriculum, they described weekly one-hour sessions in the surgical skills labs, which could serve as formative exercises. Furthermore, the structured periodic assessment strategies applied primarily included OSATS. Results found that through these weekly formative exercises, trainees exhibited a 10%-27% higher score in their OSATS when compared to previous years.

Discussion

The development of training curricula should ideally be based upon the needs of the system while keeping in line with the socioeconomic and cultural requirements of the community it is meant to serve. This not only comprises the subject matter and strategies applied to teach said material but also involves the assessment protocols to be followed in order to ensure that the objectives of the program are met. Though more emphasis is often placed upon the assessments "of" learning, in the lieu of high-stakes summative assessments, the focus of this study has been primarily on the assessments "for" learning (formative assessments) and how such assessments have been implemented in the surgical specialty of urology. As mentioned earlier, in the literature search, it was surprising to find that there were no original articles addressing this exact question. However, the studies included were more focused on the effectiveness of certain assessment tools, with formative potential.

The quality of a literature review is dependent upon the quality of the literature being critically appraised. Considering the scarcity of literature pertaining to the subject matter, only a small number of papers were encountered, with not much commonality. This imparts a high heterogeneity (both methodological and clinical) in this study.

A recurring theme, which was identified in this review, was that of the "competency" being assessed by the various exercises/models utilized in each individual paper. Of the 10 included studies, four were more directly related to technical competencies, three toward nontechnical, and three were designed to assess for both (Table 1).

Technical Skills

There is a clear emphasis on the integration of workplace-based assessments (WPA) for the purpose of assessing technical skills as this allows for the evaluation of the candidate in the clinical setting. Though the findings of the literature search suggested an overall favorable appreciation of the utility of such tools, for both formative and summative purposes, there were some factors that may influence the feedback being offered by them.

The first of these involves the burden of WPA (such as case-based discussions, mini-Clinical Evaluation Exercise/miniCEX, DOPS, and multisource feedback/MSF) on the clinical infrastructure. WPA are often time-consuming and are not often provided with dedicated allotted time in clinical practice. This may be particularly cumbersome in centers that encounter a high turnover or a busy clinic or operating room setting and may potentially lead to a restraint on the availability of the assessors’ time and attention. To remedy this restriction, Swayamprakasam et al. [31] suggested the involvement of multiple assessors to distribute this workload among them. This harkens to the General Medical Council's (GMC’s) ideal of having clinical supervisors overseeing the candidate over a specific period and assessing them for the task at hand. Further inclusion of non-medical assessors may increase the assessor pool, as in the MSF model.

Furthermore, WPA may also offer an administrative burden, especially as some assessors may not be trained in either conducting the assessment or, more importantly, not properly trained in offering structured feedback [32]. Thus, there must be an emphasis on the institution to adequately train their teaching faculty in these facets of postgraduate education.

Lastly, a factor that is often overlooked is the enthusiasm of the assessor in WPAs. Both Menon et al. [33] and Nisar et al. [34] identified that low enthusiasm during the assessment activity may influence the quality of the feedback being offered to the trainee. This may, in turn, result in a lower level of engagement of the candidate toward the activity and should be addressed.

Nontechnical Skills

Nontechnical competencies involve various cognitive and interpersonal skills, which complement practical and technical competencies. These are vital behavioral factors, which are important in decision-making, leadership, and team working. There are numerous validated scales for assessing the nontechnical skills in surgical training. Some of the more popular ones include NoTSS (nontechnical skills for surgeons), NoTECHS (nontechnical skills), and OSATS [35]. In this review, a tool called NoTSUS was identified, which is essentially a more urology-specified adaptation of the NoTSS scale [21].

NoTSS provides a system designed to offer feedback on nontechnical skills and offers a structured framework to observe and rate the behavior of a candidate in the operating room. Candidates are assessed under four major domains: situational awareness, decision-making, interpersonal skills and teamwork, and leadership.

NoTECHS, conversely, was primarily used in the aviation industry and had been adapted to the operating room environment. There are numerous versions of the scale available, addressing more specifically the surgeon, anesthetist, and scrub nurse, respectively. Structurally, both the NoTSS and NoTECHS assessment scales focus on the same four domains.

Though both these tools are well known, valid, and reliable, Khaliq et al. [36] described that NoTSS is superior for assessing individuals, whereas NoTECHS fairs better to assess the overall team setting. NoTSUS chiefly differs from NoTSS (a validated and reliable assessment scale), in that it focuses on more urology-themed procedures, involving cystoscopy, ureterorenoscopy, and intracorporeal lithotripsy.

Both Technical and Nontechnical Skills

This is the most heterogeneous group in this review, considering that studies by Sebesta et al. [19] and de Vries et al. [23] focused on training program models, whereas Alkhayal et al. [24] focused on assessment tools. As discussed above, Alkhayal et al. [24] found that around 79% of urology programs in Saudi Arabia used and advocated the implementation of portfolio reviews in their training models. Portfolios principally involve the gathering of material, which can be utilized to identify the accomplishments of the trainee and provide evidence that the objectives have been achieved during their training period. This activity may have a formative application if the trainer/tutor periodically reviews this and helps orient the candidate’s training toward where help is required. Amsellem-Ouazana et al. [37] suggested that portfolios should include a record of operative procedures, research, and other academic accomplishments.

The D-UPS protocol, as described by de Vries et al. [23], is a Dutch simulation-based training structure involving pre-session assignment of the topic and pre-test, all the way through the subsequent simulation training, evaluation, and feedback. From what can be gathered, this protocol is not influenced by any competency-based curricular model. Rather, the authors considered this as a tool to be implemented into the overall curricula and encouraged its adoption in other centers as well.

In contrast to de Vries et al.'s study, Sebesta et al. [19] explored the perceptions of the Accreditation Council for Graduate Medical Education's (ACGME’s) milestone system in urology resident evaluations. The milestone system has been discussed in the "Results" section as well. It is essentially a competency-based curriculum that focuses on six-essential domains: medical knowledge, patient care, systems-based practice, practice-based learning and improvement, interpersonal/communication skills, and medical expertise. Even though this model has not been widely deemed as useful in the setting of this service, it has been proven to be effective in various other disciplines. The authors attributed this negative perception of the Milestones system to the relative infancy of its implementation in the urology program and to the lack of specialty-specific surveys conducted by ACGME to record feedback from such centers.

Another tool that has been discussed, which covers both technical and nontechnical skills, is the SCT. Meterissian et al. [38] described that these test formats are effective tools to gauge the decision-making capabilities of surgical trainees and have illustrated the capacity of distinguishing between juniors and senior residents in a training program. These findings are in concordance with those described by Sibert et al. [22] and Nazim et al. [27], in this review. However, there are certain limitations to the application of these tests in practice. First, considering the relatively newer advent of such exercises, they are not widely used; thus, there is neither an abundant pool of such questions readily available nor is their adequate training available for faculty to prepare for such multilayered examinations. Preparation of said questions may be a time-consuming effort for already busy clinicians. Second, there is a concern about the low reliability involved with the utility of SCTs as noted by Lineberry et al. [39]. In this paper, the authors described that considering the format of these test questions and their stems, SCTs may introduce a bias toward candidates who prefer a different approach to addressing scenarios than perceived by the examiner. To control such bias may, thus, even lead to a more potential burden for the faculty while preparing these scenarios.

In terms of assessment strategies and functionality, the GMC is very robust and well structured and offers guides and recommendations at multiple levels of training and practice. The GMC emphasizes the utility of formative assessments in its various training protocols and provides a sound structure in its design. Workplace-based assessments are also encouraged as formative tools in clinical training. At a deeper glance, one can see the integration of models of nontechnical skills like NoTSS and NoTECHS in various specialties offered by the Royal Colleges, particularly the Royal College of Surgeons of Edinburgh and the Royal College of Obstetrics and Gynaecologists [40].

The question asked in this study was how formative assessments had been utilized in the various urology training programs around the world. As mentioned, though the search results were deficient in original papers on "formative assessments" directly related to urology training programs, this project had included studies where mention of strategies with the potential to be used in this intention was apparent. In this case, it may have also been useful to include further searches into the utility of specific tools, which were identified through this search like SCTs, OSATs, portfolios, etc.

## Conclusions

It seems the literature is currently more concerned about summative assessments than the formative variety. This may be due to the relative ease of collecting documented data from the former. Assessment for learning is just as important in clinical practice; however, there is an obvious deficiency in the literature base for research into this topic. Based on these findings, an increase in academic interest in the application of formative assessments in surgical training (specifically, urology) would thus be recommended.

Faculty and supervisors need to be encouraged to open dialogue with the candidate to decide upon intended goals and how to achieve them with more personalized timelines. Involvement of paramedical staff, patients, and community representatives, in the form of feedback, will also provide a better understanding of their nontechnical skills and make them aware of their strengths and weaknesses in this aspect. Furthermore, such recorded feedback may also be useful in improving the overall standard of training within the institution as well.
